# Evaluating the effectiveness and cost‐effectiveness of integrating mass drug administration for helminth control with seasonal malaria chemoprevention in Ghanaian children: Protocol for a cluster randomised controlled trial

**DOI:** 10.1111/tmi.14062

**Published:** 2024-11-27

**Authors:** Muhammed O. Afolabi, Dennis Adu‐Gyasi, Lucy Paintain, Theresa Tawiah, Mohammed Sanni Ali, Brian Greenwood, Kwaku Poku Asante

**Affiliations:** ^1^ Department of Disease Control, Faculty of Infectious and Tropical Diseases London School of Hygiene & Tropical Medicine London UK; ^2^ Research and Development Division Kintampo Health Research Centre Kintampo Ghana; ^3^ Centre for Research in Applied Biology, School of Sciences University of Energy and Natural Resources Sunyani Bono Region Ghana

**Keywords:** African children, geohelminths, integration, malaria, schistosomiasis

## Abstract

**Objectives:**

To evaluate the effectiveness and cost‐effectiveness of integrating seasonal malaria chemoprevention (SMC) with mass drug administration for helminth control among school‐aged children living in communities where the burden of malaria and helminths is high in Ghana, West Africa.

**Methods:**

This cluster randomised controlled trial will enrol 1200 children aged 5–10 years. Eligible children randomised to intervention clusters will receive SMC drugs (sulphadoxine‐pyrimethamine plus amodiaquine) and anthelminthic drugs for soil‐transmitted helminths—(albendazole), and for schistosomiasis (praziquantel), while children randomised to control clusters will receive SMC drugs alone. Pre‐ and post‐intervention blood, urine and stool samples will be collected from children in both clusters. The effectiveness of the concomitant delivery will be determined by checking whether the combination of SMC and anthelminthic drugs prevents anaemia in the children randomised to the intervention clusters compared to the children in the control clusters. Cost analysis and cost‐effectiveness of this integrated delivery approach will be determined by estimating the incremental costs and effects of co‐administration of SMC drugs with mass drug administration of anthelminthic drugs compared to SMC alone, including cost savings due to cases of moderate and severe anaemia averted.

**Expected findings:**

The findings of this study will provide evidence to inform public health recommendations for an integrated control of malaria and helminths among children living in the poorest countries of the world.

## INTRODUCTION

The changing epidemiology of malaria has shifted the burden of malaria to children aged above 5 years in many high transmission areas in sub‐Saharan Africa (SSA), with increased recognition of the consequences of malaria in school‐age children, including the detrimental effects of anaemia. This development is further aggravated by the frequency of co‐infection of malaria and helminths in many endemic settings in SSA. The geographical and epidemiological overlaps of malaria and helminth infections create an opportunity for the development and implementation of an integrated control approach for malaria and helminths [1, 2, 3].

While modest progress in the control of schistosomiasis and soil‐transmitted helminths (STH) has been achieved through mass drug administration (MDA) of albendazole (ALB) and praziquantel (PZQ) and has informed improved strategies articulated in the neglected tropical diseases (NTD) roadmaps 2030 [4], the remarkable success of seasonal malaria chemoprevention (SMC) has led to the recent World Health Organisation (WHO) recommendation for its extension to other at‐risk age groups in highly seasonal malaria transmission settings outside the Sahel region [5]. This encouraging development supports the need to explore the possibility of integrating helminth control programme with other successful delivery platforms, such as SMC, especially in countries where SMC is given to older children. Additionally, in line with the strategies of the WHO roadmap to end the neglect on NTDs, achieve the Sustainable Development Goals (SDG) 3 and 4, and ensure universal health coverage, WHO has endorsed an integrated approach of joint delivery of interventions through a common platform, such as preventive chemotherapy, integrated monitoring, evaluation and reporting [6]. Driven by the WHO recommendation of promoting the global trend towards integrated, non‐disease‐specific approaches to controlling co‐endemic diseases such as malaria and helminths [6], we demonstrated in a randomised controlled trial the feasibility, safety and acceptability of integrating MDA for schistosomiasis and STH with SMC delivery to pre‐school and school‐age children living in a co‐endemic setting in Senegal [7, 8].

To maximise the impact of our findings, we will evaluate the effectiveness of the integrated SMC and MDA of anthelminthic drugs on the prevalence of anaemia and malaria‐helminth co‐infection in a larger paediatric population than was included in the pilot trial. This study will test the hypothesis that haemoglobin (Hb) concentration will be higher in the combined SMC‐MDA group than in the SMC alone group. Given that the analysis of the economic and financial costs of delivering the integrated model will be needed to inform resource allocations, allow comparison with alternative interventions, and inform deployment of the approach, if successful, we will also evaluate the costs and cost‐effectiveness of the integrated delivery model for SMC‐MDA among the study children. The analysis will also explore how costs vary with drug prices, organisation of the delivery of the integrated approach, and how cost‐effectiveness varies depending on the differences in the effectiveness and background prevalence of anaemia [9].

## METHODS

### Study design

This will be a cluster randomised, controlled, programme‐led pragmatic study to evaluate the effectiveness of co‐administration of SMC and MDA for schistosomiasis and STH in reducing anaemia due to malaria‐helminth co‐infections among school‐age (5–10 years old) children. The trial has been registered on Clinicaltrials.gov NCT06182176 and Pan‐African Clinical Trials PACTR202312489417172. The interventions for this study are the WHO‐approved drugs for preventive treatment of malaria (sulphadoxine‐pyrimethamine [SP] + amodiaquine [AQ] [SPAQ]) [10], and helminths (ALB and PZQ) [11]. These interventions will be delivered jointly by the Ghana Health Service‐led SMC and NTD control programmes, to the children enrolled in this study. The children will be enrolled at community levels and the communities will be assigned into two equal clusters, to receive either combined SMC and anthelminthic drugs or SMC drugs only. To minimise the potential for contamination and spill‐over effect, the study communities will be separated from each other by at least 5 km. To ensure objectivity in the reporting of adverse events among the children across the two clusters, vitamin A and zinc supplement will be given to children in the control clusters randomised to receive SMC drugs only. This approach will ensure that children in the intervention and control clusters receive three drugs. Vitamin A and zinc supplements have not been shown to affect the outcomes of this study [12, 13].

### Study population

**FIGURE 1:**
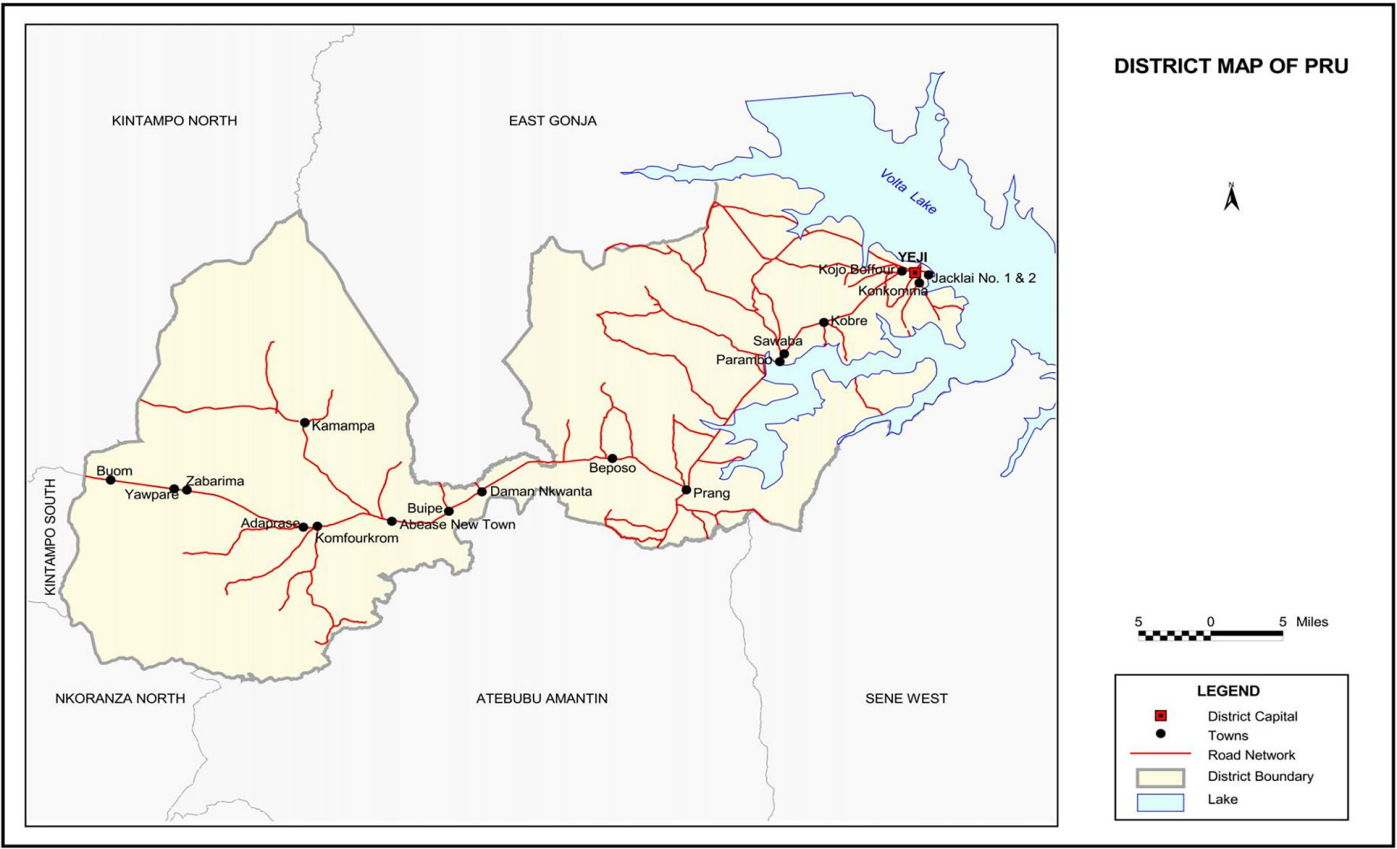
Map showing the study area in central Ghana. *Source*: Ghana Statistical Service, GIS. GIS, geometric information system.

This study will be conducted in communities within Pru East district, Bono East Region, within the forest‐savannah, transitional ecological zone in the middle belt of Ghana (Figure 1). The district experiences tropical continental or interior savannah type of climate which is a modified form of tropical continental or wet‐semi equatorial type. Major rains occur in September and October and minor rains in May and June; the annual rainfall is between 1400 mm and 1800 mm. There is a prolonged dry season between November and April. Relative humidity is 75%–80% in the wet season and 70%–72% in the dry season [14, 15, 16]. The hottest months are March and April. The main vectors for transmission of malaria are *Anopheles gambiae* and *Anopheles funestus* and malaria transmission is perennial, but peaks between April and October [17]. According to the 2022 Ghana Demographic and Health Survey, the prevalence of malaria among under‐five children in Bono East Region was 22.1% by rapid diagnostic kit test (RDT) and 12% by microscopy [18]. Similarly, the study area was reported to be endemic for schistosomiasis and STH [15].

### Sample size determination

We assumed that the mean Hb concentration in the SMC alone group will be 10.5 g/dL with a standard deviation of 1.5 g/dL [3]. Assuming an intra‐class correlation coefficient of 0.05, then 10 communities per cluster, a study of 60 children per community will have 80% power to detect an increase in mean Hb of 0.5 g/dL (to 11.0 g/dL) in the SMC plus anthelminthic group as statistically significant at 5% level. A minimum sample size of 1200 school‐age children (5–10 years) will be enrolled. This study will be implemented in three stages: pre‐intervention, intervention and post‐intervention. The pre‐intervention survey will be conducted to determine the prevalence of malaria‐helminth co‐infection in the study population.

#### Sampling for pre‐intervention stage

The Lot Quality Assurance sampling technique [19] will be used to recruit study participants in the pre‐intervention stage. For example, a total of 15 children, with a roughly even mix of boys and girls, ranging in ages from 5 to 10 years, will be randomly selected in each school within a selected community.

#### Sampling for the intervention stage of the trial

There will be two study arms in the intervention phase of this trial—intervention and control arms. Ten schools within Pru East communities will be randomised to the intervention arm and another 10 schools will be randomised to the control arm. As calculated above, a minimum of 60 children who satisfy all eligibility criteria will be enrolled sequentially in each of the 10 schools randomised into the intervention and control arm, respectively. To identify these children, we will undertake a census of all children aged 5–10 years in the communities selected for the intervention stage of the trial. Subsequently, we will use a sampling frame for random sampling from the census list to enrol the required number of children into each study arm. We will keep a reserve list of potentially eligible children in case of any refusals from the sampling list.

Data collection for the cost‐effectiveness analyses will involve semi‐structured interviews with key informants from the regional and district education and health offices, using purposive sampling to select health facility personnel, community health workers (CHWs) and school teachers involved in the delivery of SMC with or without MDA for helminths. Approximately 6–12 interviews are anticipated at the regional and district level, up to 10 with health facility personnel, 10–15 with CHWs and 10–20 with school teachers. Key informants will be chosen to reflect maximum expected variation across the study area, for example, a mix of urban and rural communities, distance from the district centre, etc. This flexible and purposive approach to sampling is a standard and important feature of economic evaluations.

### Eligibility criteria

Inclusion criteria will include male and female children aged 5–10 years whose parents/caregivers are willing to provide written informed consent and a positive assent by children aged ≥7 years (in line with legal regulations in Ghana); willingness to provide finger‐prick blood samples, urine and stool samples; residency in the study area for at least the past 6 months and willingness to be available in the study area for about 6 months of follow‐up after enrolment. Exclusion criteria will include an acutely ill child at the time of the drug administration; a child whose parent/caregiver declines to provide consent; a known human immunodeficiency virus (HIV) positive child receiving co‐trimoxazole prophylaxis; a child who has received a dose of either SP or AQ in the previous 28 days; a child who has received ALB or PZQ during the previous 6 months; and a child with a known allergy to any of the study drugs.

### Ethical considerations

Approvals for the study have been obtained from the Ethics Committees of the London School of Hygiene & Tropical Medicine (Ref: 29839), Kintampo Health Research Centre, Ghana (Ref: KHRCIEC/2024‐01) and the Ghana Health Service (Ref: GHS‐ERC: 024/11/23). Regulatory approval has also been obtained from the Ghana Food and Drug Administration (Ref: FDA/247). The trial will be externally monitored and an independent data safety and monitoring board will oversee participant safety and evaluation of the results.

### Study procedures

#### Stakeholders' engagement and community sensitisation

The study team has met with the Programme Managers and National Coordinators of the SMC and NTD programmes of the Ghana Ministry of Health, as well as with the regional and district Education directors and supervisors, to seek their collaboration and partnership in the implementation of this study. Community sensitisation and engagement meetings will be organised prior to the commencement of the study to explain the nature of the study to the parents/caregivers of potentially eligible children.

##### Pre‐intervention stage

After written informed consent has been obtained from a parent/caregiver 1 day before the planned start date of the pre‐intervention survey, along with a positive assent from children aged ≥7 years when required, a pre‐labelled stool collection container will be provided to the parent/caregiver. The parent/caregiver will be encouraged to collect stool from their child and keep this safely for the research team who will visit them the following day, when a purpose‐designed electronic questionnaire will be administered to the parent/caregiver. The questionnaire will cover information such as socio‐demographic, health and residence characteristics, history of deworming and malaria treatment. Height/length (cm) and weight (kg) of each child will be measured and anthropometric indices height‐for‐age, weight‐for‐age, weight‐for‐height and body mass index, will be calculated using the WHO AnthroPlus software (www.who.int/growthref/tools/en/). Each study participant's household will be mapped with global‐positioning system (GPS) coordinates obtained with a hand‐held GPS device. Finger‐prick blood samples will be collected from each child participant for thick and thin smear examination for malaria microscopy, and blood spot filter papers will be collected for DNA isolation and polymerase chain reaction (PCR) amplification for species determination. Hb concentrations will be measured using the Haemocue® method. Freshly voided urine samples will be collected from all study children. Urine filtration test will be used to quantify *Schistosoma(S) haemotobium*, according to standard guidelines [20]. Parallel testing for circulating cathodic antigens (CCA) will be used to complement the filtration test. The urine CCA dipstick test has been extensively used in SSA and had sensitivity and specificity values for *Schistosoma mansoni* ranging from 52.5% to 63.2% and 57.7% to 75.6%, respectively [21]. Stool samples will be collected to detect intestinal helminths. Duplicate Kato‐Katz thick smears will be prepared from the stool samples and examined by experienced technicians using light microscopy to determine the egg counts for *S. mansoni* and STH. The numbers of eggs per slide will be used to obtain a measure of the number of helminth eggs per gramme of faeces (EPG). The intensity of the helminth infection will be categorised according to standard guidelines [20]. A multiplex PCR assay will also be used for simultaneous detection of mixed infections of helminths [22]. Quality control will be performed by re‐examining at least 10% of randomly selected blood slides, urine filters and Kato‐Katz smears by an experienced independent laboratory scientist. Laboratory staff performing the analyses will be masked to the origin of samples.

##### Intervention stage

Given that the focus of this project is to evaluate the effectiveness of the integrated SMC‐MDA control programmes, the first day of starting the intervention stage (Day 0) will correspond to the day prior to the commencement of the first cycle of SMC in the study area. On Day 0, the communities will be randomised at community level into two clusters at 1:1 ratio to receive either combined SMC and anthelminthic drugs (intervention cluster) or SMC drugs only (control cluster). Given that 4‐aminoquinoline drugs are reported to have antagonistic pharmacological interactions with PZQ [23], the SMC partner drugs, AQ (a 4‐aminoquinoline derivative) and SP will not be administered with anthelminthic drugs (ALB and PZQ) on the same day in this study. Therefore, eligible children randomised into the intervention clusters will receive ALB and PZQ on Day 0 and those randomised into the control clusters will receive vitamin A and zinc supplement as control drugs on this day. On the following day, which corresponding to the start (Day 1) of the first SMC cycle, the children in the control and intervention clusters will receive SPAQ according to the WHO and Ghana SMC implementation guidelines. This will be followed on the second and third day of the SMC cycle with all study children in the two groups receiving AQ, in line with the SMC guidelines [5]. The study will administer AQ on Days 2 and 3 to the children in the presence of their parents/caregivers. Trained field workers will visit all enrolled children daily, irrespective of the study arms, at home starting from the evening of Day 0 till 3 days after completion of the first cycle of SMC course, using a purpose‐designed electronic diary card, to collect adverse drug reactions and adverse events such as vomiting, skin rash, body weakness, etc. Trained research assistants will visit each child 1 month after each round of SMC cycle to check that there are no severe reactions to the previous treatment and to give the next round of treatment (Table 1).

**TABLE 1:** Schematic diagram of the study procedures for the effectiveness study.

SMC cycle	1					2, 3 and 4	End of malaria season (i.e., 1 month after completion of the last SMC cycle)
Timelines (days)	Pre‐intervention survey	Day 0	Day 1	Days 2 and 3	Days 4–6		Post‐intervention survey
Study procedures						Passive surveillance involving collection of cases of breakthrough clinical malaria during the monthly cycle of SMC drugs administration according to WHO guidelines till the end of the malaria season	
Informed consent ± assent	X						
Enrolment and questionnaire administration to parents/caregivers	X						
Distribution of collecting bottles for stool sample a day preceding sample collection day.	X						
Sample collection (finger‐prick blood, urine, stool samples)	X	X					
Randomisation and intervention		X					
Intervention villages: SMC + anthelminthics		ALB + PZQ	AQ + SP	AQ			
Control villages: SMC + placebo		Vit A + Zn	AQ + SP	AQ			
Safety assessments (active surveillance)		X	X	X	X		
Post‐intervention survey							X
Sample collection: Finger‐prick							X
Sample collection: stool							X
Sample collection: urine							X

Abbreviations: ALB, albendazole; AQ, amodiaquine; PZQ, praziquantel; SMC, seasonal malaria chemoprevention; SP, sulphadoxine‐pyrimethamine Vit A, vitamin A; Zn, zinc.

##### Post‐intervention stage

All child participants enrolled in the intervention study will be evaluated 1 month after the last course of SMC, which corresponds to the end of malaria transmission season, to measure their Hb concentration and to obtain a further blood film, dried blood spot, stool and urine samples, to test for malaria‐helminth co‐infections using parasitological methods such as microscopy, Kato‐Katz, urine filtration test and improved diagnostic tools including molecular methods (quantitative polymerase chain reaction), as described above.

##### Data collection for the cost and cost‐effectiveness analyses

The incremental financial and economic costs of delivering the integrated SMC‐MDA programme in the intervention stage of this study will be estimated from a provider (Government of Ghana and donor) perspective, and compared to the counterfactual of giving SMC drugs alone. Financial cost data (direct expenditure on intervention delivery) and economic costs (donated goods or services, such as time of salaried health workers and schoolteachers) will be collected through review of project records and semi‐structured key informant interviews (KIIs) at district, health facility, CHW and school levels. Detailed costs will be captured for disaggregated cost categories, including SMC and MDA drugs; drug supply chain; school teacher delivery of doses; supervision; training; planning meetings; sensitisation; and supplies. Incremental costs required for the integrated delivery of MDA with SMC above what would be required for SMC delivered alone will be identified. The cost of diagnosis and treatment of moderate and severe anaemia from the health service perspective will be estimated using a combination of primary and secondary data. Cost per outpatient visit and cost per inpatient day (excluding diagnosis and treatment) will be obtained from existing data sources [24, 25]. Costs of diagnostic tests, medicines and provider time for treating cases of moderate and severe anaemia will be captured through KIIs with health staff at facilities providing outpatient and inpatient services in the study area. These parameters will be used to estimate incremental cost savings due to cases of moderate and severe anaemia averted by SMC plus MDA compared to SMC alone.

**FIGURE 2:**
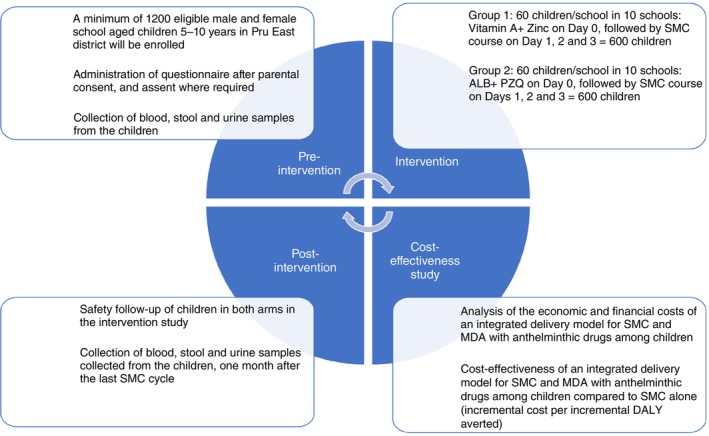
Schematic design of the study. DALY, disability‐adjusted life‐years; MDA, mass drug administration; SMC, seasonal malaria chemoprevention.

For the cost‐effectiveness analysis (CEA), the main measure of the effects will be disability‐adjusted life‐years (DALYs) averted. DALYs combine mortality (years of life lost [YLLs]) and morbidity (years lived with disability [YLDs]) into a single measure of effect, allowing the impact of SMC with MDA to be compared with the impact of different types of health interventions. The effect measure used to calculate DALYs will be moderate and severe anaemia cases averted (where anaemia as the primary outcome for the CEA, which is where the main incremental health benefit from co‐delivery is expected), derived from unadjusted results of the trial, calculated on the basis of intention‐to‐treat (ITT). DALYs will be modelled using a 3% discount rate in the base case, no age weighting, standard DALY weights and life tables [26], and other secondary data (e.g., duration of an episode of anaemia) [27]. The CEA results will be expressed as an incremental cost‐effectiveness ratio (ICER). The ICER is calculated as the difference in costs between MDA + SMC versus SMC alone, divided by the difference in effects between those strategies (Figure 2).

$$\text{ICER}=\frac{\text{cost of administering}\;\mathrm{MDA}+\mathrm{SMC}\text{(intervention)}-\text{cost of administering}\;\mathrm{SMC}\text{(control)}}{\left(\text{DALY averted in intervention}\;\mathrm{arm}-\text{DALYs averted in control}\;\mathrm{arm}\right)}$$

##### Endpoints

The primary trial endpoint will be a change in Hb concentration, measured by HemoCue®, on the day of inclusion, and post‐intervention at the end of malaria transmission season, that is, 1 month after the last SMC cycle. The secondary endpoints will include incidence of clinical malaria, defined as fever of >37.5°C or a history of fever in the preceding 48 h, and a positive malaria blood film of any parasite density, detected by passive case detection during the surveillance period; change in prevalence of anaemia between the day of inclusion and post‐intervention at the end of malaria transmission season (anaemia will be defined as an Hb less than 11 g/dL); the incidence of solicited adverse events and adverse drug reactions assessed to be related to the study medications during a period of six consecutive days after administration of study drugs; prevalence and density of *Plasmodium falciparum* infection at the post‐intervention survey; prevalence and density of helminth infection; and prevalence and density of malaria‐helminth co‐infection at the post‐intervention survey. Tertiary endpoints will include the cost per child treated per year and the cost per anaemia case averted calculated by a cost analysis, and the incidence of anaemia among the children in the intervention and control arms will be used to estimate the incremental cost‐effectiveness of integrated SMC plus anthelminthic treatment versus SMC alone, in terms of incremental economic costs per DALY averted (Box 1).

BOX 1 **Study medications.**

Given that this study will be implemented during an SMC campaign and in collaboration with the SMC programme of the Ghana Ministry of Health, the SMC drugs will be obtained from the SMC implementation unit of the Ministry of Health. The ALB and PZQ for this study will be procured from GlaxoSmithKline and Merck, respectively. Vitamin A will be a liquid, oil‐based preparation of retinyl palmitate or retinyl acetate, obtained from the United Nation's Children Fund, Ghana office. The zinc preparation will be a citrus‐flavoured tablet containing 25 mg Zn in the form of zinc sulfate and will be obtained from Biolectra Zink; Hermes Arzneimittel GmbH, Munich, Germany. To minimise under‐and over‐dosing while keeping simple dosage recommendations, the doses of SP and AQ for SMC, anthelminthics, vitamin A and zinc supplement will be based on the child's age, as follows:

- SP tablet (500 mg sulphadoxine + 25 mg pyrimethamine): Children aged 5–10 years will receive two tablets as a single dose on the first day.
- AQ tablet (153 mg base): Children aged 5–10 years will receive two tablets as a single dose for 3 consecutive days.
- ALB tablet (200 or 400 mg depending on age): Children aged 5–10 years will receive a single dose of ALB 400 mg.
- PZQ tablet (600 mg): Children aged 5–10 years will receive PZQ based on their body weight of 40 mg/kg.
- Vitamin A oral liquid (200,000 IU): Children aged 5–10 years will receive 200,000 IU vitamin A.
- Zinc tablet (40 mg/kg): Children aged 5–10 years will receive zinc supplement based on their body weight of 40 mg/kg.

The medications will be administered to the study children by a member of the SMC and NTD control programme staff in the presence of the study investigator and parents/caregivers of the children. The investigator will ensure that blood, urine and stool samples have been collected from the child before administration of the study drugs.

Given that the toxicities caused by interactions of PZQ with ALB are dose‐dependent and can be reduced by taking PZQ with carbohydrate meals [28], PZQ will be given to the children based on the dose recommended by WHO, which is dependent on the body weight of the child (40 mg/kg). We will ensure that the children take carbohydrate‐rich meals by providing them healthy snacks before the administration of the drugs. If the child vomits, we will wait for 30 min and then try again. If vomiting occurs a second time, the medication will not be repeated, and the child will not be enrolled in the study. If the drug‐causing problems are ALB or PZQ, then the child will continue to receive the SMC drugs. If a child misses one of the SMC drugs at a round, the child will continue to receive further doses, in line with the SMC implementation guidelines. Children who start treatment but could not be found at home after reasonable efforts will be excluded from the study. A simple, user‐friendly recording tool will be used to capture the drug administration for each child.

### Statistical analysis plan

The field and laboratory data will be merged, harmonised, cleaned and analysed with STATA® version 18.1 SE (Stata Corp., College Station, TX, USA). Descriptive statistics such as frequencies and proportions will be used to summarise the baseline parasitic parameters and socio‐demographical characteristics of the study participants by cluster arm. Data will be analysed using an ITT approach. The proportions of adverse events and the incidence (average number per participant) of reported adverse events will be compared between the study groups using the Chi‐squared test. Logistic regressions with robust standard errors will be used to estimate odds ratios and 95% confidence intervals. Wald tests will be used to assess the evidence of difference in odds between study groups, after controlling for baseline characteristics (sex, age group, weight and malaria and helminth infections' occurrence). Effect modification between intervention period and study groups will also be investigated using the Wald test. The geometric means of the *P. falciparum*, STH and schistosome intensities will be estimated and plotted before and after intervention periods. A univariable Poisson regression with robust standard error and Wald test will be used to compare the pre‐intervention parasites intensities across groups, and a multivariable Poisson regression including the pre‐intervention parasites intensities as a covariate, adjusting for other baseline characteristics described above, and Wald test will be used to compare the post‐intervention parasites intensities. The proportions of adverse events and the incidence (average number per participant) of reported adverse events will be compared between the study groups using the Chi‐squared test. All statistical tests will be two‐sided at a significance of *α* <0.05.

The economic analysis will have three parts:

- a detailed cost analysis of (a) the incremental financial and economic costs of implementing integrated treatment versus SMC alone; (b) modelling costs of delivery at scale; and (c) cost savings from cases of anaemia averted;
- a budget analysis that compares the net financial costs of delivery at scale minus cost savings from anaemia cases averted against the appropriate Ministry of Health budget to assess if the new intervention is affordable; and
- a CEA that uses a decision model approach to estimate the ICER of integrated SMC + anti‐helminthic treatment versus SMC alone, in terms of incremental economic costs per incremental measure of effect (DALY averted).

The impact of uncertainty and heterogeneity on results will be explored using deterministic and probabilistic sensitivity analyses to assess the robustness of ICERs to key model parameters (e.g., cost of drugs, effectiveness measures, time taken to deliver MDA in addition to SMC, etc.). The ICER per DALY averted will be compared against context‐appropriate willingness‐to‐pay thresholds [29, 30].

## DISCUSSION

Malaria‐helminth co‐infection is prevalent among children living in SSA, due to complex interactions of environmental and host factors that support the co‐existence of *Plasmodium* species with parasitic helminths, including STH and *Schistosoma* species [31, 32]. The geographical overlap in the co‐endemicity of helminths and malaria parasites may result in synergistic or antagonistic interactions between helminth and malaria parasites [32, 33, 34]. The additive effects of malaria‐helminth co‐infection on Hb concentration probably contribute substantially to mortality from malaria in children in SSA [1, 35]. Separate vertical implementations of SMC and MDA for helminth control have had limited impact on the prevalence of anaemia [1, 2, 3]. This, therefore, creates an opportunity to exploit the overlap of malaria and helminth to develop and evaluate an integrated approach to achieve effective control of malaria and helminths in endemic settings. This study builds on a previous study that established the safety, tolerability, feasibility and acceptability of the integrated approach. The current trial will address the knowledge gap on the impact and cost‐effectiveness of co‐administration of SMC and anthelminthic drugs on the prevalence of anaemia and malaria‐helminth co‐infection among vulnerable children in a high‐burden country.

The current study was designed and will be implemented in partnership with the Ghana Health Service to facilitate adoption of the integrated SMC‐MDA model and scale‐up if the intervention is found to be effective. In addition, the secondary objectives will inform the deployment of the integrated approach if the intervention is predicted to be cost‐effective and affordable at scale. Findings from this study could have important policy and practice implications for malaria and helminth control programmes across SSA.

## FUNDING INFORMATION

This study is implemented as part of a career development fellowship awarded to MOA, which is funded under the UK Research and Innovation Future Leaders Fellowship scheme (MR/X023133/1). The funder had no role in the study design, data collection and analysis, decision to publish or preparation of the manuscript.

## CONFLICT OF INTEREST STATEMENT

Authors declare no conflict of interest.
